# Relationship between the *IL23R* SNPs and Crohn’s Disease Susceptibility and Phenotype in the Polish and Bosnian Populations: A Case-Control Study

**DOI:** 10.3390/ijerph16091551

**Published:** 2019-05-02

**Authors:** Krzysztof Borecki, Iwona Zawada, Nermin Nusret Salkić, Beata Karakiewicz, Grażyna Adler

**Affiliations:** 1 Department of Studies in Antropogenetics and Biogerontology, Pomeranian Medical University, Żołnierska 48, 71-210 Szczecin, Poland; k.borecki.pum@gmail.com; 2 Department of Gastroenterology, Pomeranian Medical University, Unii Lubelskiej 1, 71-252 Szczecin, Poland; iwonazaw@wp.pl; 3 Department of Gastroenterology and Hepatology, University Clinical Center Tuzla, Trnovac bb, 75000 Tuzla, Bosnia and Herzegovina; snermin@gmail.com; 4 Department of Public Health, Pomeranian Medical University, Żołnierska 48, 71-210 Szczecin, Poland; beata.karakiewicz@pum.edu.pl

**Keywords:** Crohn’s disease, *IL23R*, SNPs

## Abstract

It is suggested that IL-23/IL-17 axis and single nucleotide polymorphisms (SNPs) of *IL23R* may have crucial role in pathogenesis of Crohn’s disease (CD). Thus, we sought to assess the *IL23R* SNPs contribution to susceptibility and phenotype of CD. We recruited 117 CD subjects and 117 controls from Poland and 30 CD subjects and 30 controls from Bosnia and Herzegovina (B&H). Two common *IL23R* SNPs: rs1004819, rs7517847 were genotyped using TaqMan SNP assays. In the Polish population it was found that allele rs1004819: A increases the risk of CD, while allele rs7517847: A is protective against disease development. In Poles the co-carriage of two *IL23R* risk genotypes was associated with increased risk of CD. A significantly increased risk of CD early onset was observed in Poles carrying at least one rs7517847: G allele. It was also found that *IL23R* SNPs may be associated with structuring/penetrating CD behavior, as alleles rs1004819: A and rs7517847: G were significantly less frequent in patients without complications, from Poland and B&H, respectively. Allele rs1004819: A was also significantly more frequent in Poles with penetrating CD. These results confirm *IL23R* SNPs contribution to CD susceptibility in the Polish population and suggest their impact on early age of onset and more severe disease course.

## 1. Introduction

Crohn’s disease (CD) is an incurable chronic autoimmune disorder determined by environmental, immunological and genetic factors, characterized by remitting and relapsing inflammation that may occur anywhere along the length of the gastrointestinal tract (GIT) [1,2].

Recently, the crucial role of the interleukin 23 (IL-23) signaling pathway (IL-23/IL-17 axis) in CD pathogenesis has been suggested. According to the leading hypothesis the chronic intestinal inflammation is stimulated by pro-inflammatory cytokines including IL-17A, IL-17F, IL-21, and IL-22 produced by activated Th17 (CD4+) lymphocytes [3,4]. The essential role of IL-23 and Th17 lymphocytes activation for the induction of chronic intestinal inflammation was demonstrated in animal models [5,6]. It was also showed that Th17-related cytokines: IL-17 and IL-22 serum levels are increased in CD and correlates with disease activity [7,8,9,10]. Several studies have reported an elevated expression of IL-23, Th17 cells, and Th17-related cytokines in inflamed mucosa of CD patients [9,10,11,12]. In the study by Annuziato et al. an increased numbers of IL-17 producing T cells (CD4+) was found in the disease-affected gut areas of CD patients, compared to their peripheral blood or healthy gut areas of subjects without CD [13].

The *IL23R* gene (GenBank: NM_144701, GeneID: 149233) is located on chromosome 1p31 and contains at least 11 exons [14,15]. Apart from the Th17 lymphocytes its expression and sensitivity to the IL-23 has been observed in multiple cell lines, including macrophages, neutrophils, NKT, and dendritic cells [16]. To date several single nucleotide polymorphisms (SNPs) of *IL23R* have been linked with CD susceptibility in European-descent populations [17]. Two of them, rs1004819 G > A and rs7517847 T > G showed a strong association with CD in genome-wide association studies (GWAS) and subsequent case-control studies [15,17,18,19]. It was also suggested that rs1004819: A allele is a risk factor for CD, while rs7517847: G allele confers protection against disease development [17,20,21]. The rs1004819 and rs7517847 SNPs are located in non-coding regions of the *IL23R* gene (intron 5 and 6, respectively) and its influence on the function of *IL23R* remains uncertain [17,22]. However, it is supposed that due to intronic localization they could regulate the alternative splicing of the premature *IL23R* mRNA, resulting in the generation of a different IL-23R protein isoforms [23,24,25].

One of the most promising therapeutic approach in CD is based on the monoclonal antibodies targeting IL-23 (p40 and p19) subunits [26]. However, it is suggested that the direct inhibition of IL-23 receptor signaling via IL-23 receptor subunit antagonists or IL-23R competitive inhibitors could potentially provide a higher therapeutic value [27].

Therefore, given the IL-23 and *IL23R* biological and potential therapeutic significance in CD, the investigation of the *IL23R* SNPs gene may be beneficial for the optimization of the future therapeutic strategies. Here we aimed to assess the contribution of the *IL23R* SNPs in determining susceptibility and phenotype of CD in population from Poland and Bosnia and Herzegovina (B&H).

## 2. Materials and Methods

### 2.1. Study Participants

This study was approved by local Ethics Committees (decision reference numbers: Poland: KB-0012/131/15 and KB-0012/90/17; B&H: 29-BS-4329/11). All study subjects provided written informed consent before the enrolment.

We recruited 117 CD patients and 117 controls from Poland and 30 CD patients and 30 controls from B&H. Crohn’s disease diagnosis was determined according to the clinical, radiological, endoscopic, and histological criteria [28]. Subjects enrolled as controls have had CD excluded with colonoscopy but underwent diagnostic testing for reasons other than inflammatory bowel disease (IBD). Demographic and clinical data were obtained directly from patients or from their medical records. Phenotypic data of genetically analyzed patients were available for 99 Polish CD patients and all 30 CD patients from B&H. Disease phenotype was classified according to Montreal Classification (2005) [29]. The demographic and clinical details of the Polish and Bosnian CD subjects and controls are shown in Table 1.

### 2.2. DNA Extraction and Genotyping

In total, 294 whole blood samples were collected from CD subjects and controls from both populations. Genomic DNA was isolated from peripheral blood leukocytes by standard procedures using the QIAamp DNA Mini Kit (Qiagen, Hilden, Germany). The *IL23R* rs1004819 and rs7517847 SNPs genotyping was performed using LightCycler 96 Real-Time PCR System (Roche Diagnostics, Warsaw, Poland), TaqPath ProAmp Master Mix (Thermo Fisher Scientific, Waltham, MA, USA) and commercial pre-designed TaqMan SNP assays (rs1004819: C_1272321_10, rs7517847: C_30369702_10; Thermo Fisher Scientific, Waltham, MA, USA), according to the manufacturer’s instruction. Samples were first heated for 10 min at 95 °C, before amplification as follows: 40 cycles of two-step PCR program at 95 °C for 15 s and 60 °C for 1 min.

### 2.3. Statistical Analysis

The *IL23R* genotype and allele frequencies were determined by direct counting. Deviations from Hardy-Weinberg equilibrium (HWE) were estimated using a chi-square (χ^2^) test from R package “HardyWeinberg” (v. 1.5.9). Overall differences in genotype distributions between CD patients and controls from both studied populations were evaluated using χ^2^ test. For the statistical analysis of the *IL23R* SNPs genotypes and alleles association with CD susceptibility and phenotype Fisher’s exact test was applied. Above mentioned statistical analyses were carried out using GraphPad Prism v. 5.03 software (GraphPad Software Inc., San Diego, CA, USA). All *p* values were two-sided and considered statistically significant at *p* < 0.05.

## 3. Results

In studied populations, all analyzed SNPs were in HWE, both in CD subjects and controls. The genotype and allele frequencies are shown in Table 2. Statistical analysis revealed a significant differences in the overall distribution of *IL23R* rs1004819 and rs7517847 genotypes between the Polish CD patients and controls (*p* = 0.0093 and *p* = 0.0129, respectively). There were no significant differences in the overall distribution of both *IL23R* SNPs genotypes between the Bosnian CD patients and controls (*p* > 0.05). The association of *IL23R* genotypes with CD was analyzed using co-dominant, dominant and recessive genetic models of inheritance. A significant association between rs1004819 SNP and CD risk was found in the Polish population, under dominant (*p* = 0.0058; OR (95% CI) = 2.15 (1.27–3.62)) and both co-dominant (A/A vs. G/G and G/A vs. G/G: *p* = 0.0142; OR (95% CI) = 3.19 (1.27–8.02) and *p* = 0.0188; OR (95% CI) = 1.95 (1.13–3.39)) genetic models. In Poles, a significant associations between the rs7517847 SNP and protection against CD was found under dominant (*p* = 0.0323; OR (95% CI) = 0.54 (0.32–0.92)), recessive (*p* = 0.0195; OR (95% CI) = 0.36 (0.16–0.82)) and a single co-dominant (G/G vs. T/T: *p* = 0.0064; OR (95% CI) = 0.28 (0.12–0.68)) genetic models. Allelic analysis revealed that rs1004819: A allele was significantly over-represented in the Polish CD group compared to controls (*p* = 0.0028; OR (95% CI) = 1.85 (1.25–2.75)), while allele rs7517847: G was significantly more frequent in the Polish controls than CD subjects (*p* = 0.0054; OR (95% CI) = 0.57 (0.39–0.84)). There were no significant differences in the genotype and allele frequencies of both *IL23R* SNPs between the Bosnians CD patients and controls (*p* > 0.05).

Considering that both SNPs were related with CD susceptibility in Poles, the joint effect of the combined risk genotypes of rs1004819 and rs7517847 SNPs on the risk of CD was investigated. Individuals from both populations were grouped on the basis of number of carried risk genotypes. The effect of *IL23R* SNPs on CD development was assessed separately for homozygous (rs1004819: A/A and rs7517847: G/G) and homo- and/or heterozygous risk genotypes (rs1004819: A/A or G/A and rs7517847: G/G) co-occurrence. The frequencies of the *IL23R* genotypes co-occurrence in CD patients and controls from both population are shown in Table 3. The results of the statistical analysis showed a significant over-representation of homozygous (*p* = 0.0444; χ^2^ = 6.23; df = 2) and homo- and/or heterozygous (0.0041; χ^2^ = 10.98; df = 2) risk genotypes in the Polish CD group compared to controls. The carriage of one or two of the homozygous risk genotypes (rs1004819: A/A or/and rs7517847: G/G) was significantly more frequent in the Polish CD patients than controls (1 vs. 0 and 2 vs. 0: *p* = 0.0128; OR (95% CI) = 2.23 (1.19–4.17) and *p* = 0.0034; OR (95% CI) = 2.76 (1.44–5.31), respectively). In addition, the carriage of two homo- and/or heterozygous *IL23R* risk genotypes (rs1004819: A/A or G/A and rs7517847: G/G) was found to be significantly more frequent in the Polish CD patients (2 vs. 0: *p* = 0.0309; OR (95% CI) = 2.71 (1.10–6.69)). In the Bosnian population, there were no significant differences in the occurrence of combined *IL23R* risk genotypes in CD patients compared with controls (*p* > 0.05).

The association of *IL23R* SNPs with disease sub-phenotypes was also investigated. Patients were grouped based on their age of CD onset, disease location and clinical behavior according to the Montreal classification. *IL23R* rs1004819 and rs7517847 genotype and allele distributions were compared between subgroups of CD patients with and without given categories. In the statistical analysis the combined counts of the minor allele containing genotypes (rs1004819: A/A + G/A and rs7517847: G/G + G/T) were used. The genotype and allele frequencies in subgroups of the Polish and Bosnian CD patients and association between genetic variants of *IL23R* and CD phenotype are presented in Table S1. Results showed a significant over-representation of genotypes with at least one rs7517847: G allele (G/G + G/T vs. T/T: *p* = 0.0309; OR (95% CI) = 9.14 (1.10–76.19)) in the Polish subjects with early CD onset (A1) compared to patients with adult disease onset (A2 or A3). It was also found that allele rs1004819: A was significantly less frequent (A vs. G: *p* = 0.0136; OR (95% CI) = 0.48 (0.27–0.86)) in the Polish subjects with non-stricturing, non-penetrating CD (B1) compared to subjects with complications (B2 or B3). At the same time, allele rs1004819: A was significantly over-represented (A vs. G: *p* = 0.0234 OR (95% CI) = 2.22 (1.13–4.39)) in the Polish subjects with penetrating CD (B3) compared to subjects with non-penetrating non-stricturing or structuring disease phenotype (B1 or B2) (A vs. G: *p* = 0.0234 OR (95% CI) = 2.22 (1.13–4.39)). For the group from the Bosnian population, allele rs7517847: G was observed significantly less frequently (G vs. T: *p* = 0.0089; OR (95% CI) = 0.2105 (0.07–0.63)) in subjects with non-stricturing non-penetrating CD (B1) than subjects with CD complications (B2 and/or B3). Results revealed no significant associations between both of the investigated *IL23R* SNPs and age at diagnosis of CD in the Bosnian patients (*p* > 0.05). In both studied populations no significant associations between the *IL23R* SNPs and CD location were found (*p* > 0.05).

## 4. Discussion

The incidence of IBD and particularly CD are recently rising in developing countries [30,31]. Although, the precise aetiopathogenesis of CD remain elusive, it has been suggested that *IL23R* gene polymorphisms could contribute to susceptibility and phenotype of disease [17,32,33,34]. The data on the *IL23R* rs1004819 and rs7517847 SNPs frequencies in CD subjects from Poland are limited, and in Bosnia and Herzegovina are non-existent. Thus, in this study the association of two *IL23R* SNPs with CD was investigated in above mentioned populations. Additionally, the correlation between *IL23R* SNPs and disease clinical manifestation defined according to the Montreal classification was evaluated.

Both of the investigated *IL23R* SNPs were associated with CD susceptibility at the genotype and allele level in the Polish, but not in the Bosnian population. In Poles the *IL23R* rs1004819: A variant has been found to be associated with increased risk of CD, while the rs7517847: G allele showed protection against disease development. These results are consistent with findings of the previous studies on European-descent populations. The association of rs1004819 and rs7517847 with CD was reported in German population by Glas et al. Similar to our results the rs1004819: A allele was shown to be a CD risk factor and the rs7517847: G allele was found to be a protective variant [34]. The *IL23R* rs1004819: A variant association with higher CD risk was also reported in two British studies [35,36]. The minor allele of rs1004819 was also found to be associated with an increased CD risk in the Spanish [22], Australian [33], Finnish [37] and Swedish populations [38]. On the other hand, no association of rs1004819: A with CD was found by Doecke et al. in the New Zealand cohort [33]. Additionally, no significant association of *IL23R* rs1004819 SNP was found in another Polish study by Jakubowska-Burek et al. Interestingly, in their study, the heterozygous risk genotype rs1004819: G/A over-representation was observed in control group, while the rs1004819: A/A genotype frequency was higher in CD subjects [39]. Similar genotypes distribution, with higher frequency of the heterozygous risk genotype rs1004819: G/A in controls compared to CD patients and without significant association with CD, was also observed in our study, in the Bosnian population.

A strong association of the rs7517847 SNP with an increased risk of CD was reported by Newman et al. in non-Jewish cohort from the Canadian population [40]. The allelic association of the rs7517847 was also replicated in the Spanish population, by Oliver et al. [22]. The *IL23R* rs7517847 significant association with CD in the dominant genetic model as well as the rs7517847: G variant protective effect for disease development were found in the Slovenian [41], Italian [42] and another Spanish study [43]. For the Italian population, contradictory results were reported by Lauriola et al., as the rs7517847 minor allele frequency (MAF) was found to be increased in subjects with CD (*n* = 19), compared to controls (*n* = 20) [44]. Comparable results, but without statistical significance, were obtained in our study in Bosnian cohort. On the other hand, in the recent Italian study on large group of CD patients (*n* = 708) and controls (*n* = 537), the *IL23R* rs7517847: G variant was found to be significant protective against CD development [45]. Similar results were obtained in the New Zealand [32,33], Australian [33] and British populations [35]. In the Polish study by Jakubowska-Burek et al. a significantly higher frequency of genotypes containing rs75717847: T risk variant (T/T and T/G) was observed in CD subjects compared to controls. However, no significant differences were found in the proportion of genotypes containing protective *IL23R* rs75717847: G variant (T/T and T/G) [39].

It is worth mentioning that contradictory results of the *IL23R* SNPs association with CD were reported in non-European populations. In Brazilians *IL23R* rs1004819: A allele was associated with CD, while no association was found for rs7517847 [46]. At the same time, no significant differences in genotypes distribution of both *IL23R* SNPs were revealed in the Japanese study by Yamazaki et al. [47]. The lack of rs1004819 association with disease susceptibility was also described in Malaysians [48]. Thus, as it was shown in the meta-analysis by Xue et al., the *IL23R* rs1004819 and rs7517847 SNPs are associated with CD mainly in populations of Caucasian origin [17].

The results of this study also show that co-occurrence of homozygous and homo- and/or heterozygous *IL23R* risk genotypes may increase the risk of CD development, as it was significantly more frequent in the Polish CD subjects than controls. Therefore, it is possible that *IL23R* risk variants could have an additive effect on the risk of CD. Due to small sample size, we failed to replicate these results in the Bosnian cohort.

Additionally, the analysis of *IL23R* SNPs correlation with CD clinical manifestation revealed a significant over-representation of genotypes with at least one rs7517847: G allele (G/G and G/T) in those Polish individuals with early CD onset (A1) as compared with patients with adult disease onset (A2 or A3). The results also showed a significantly lower MAF of rs7517847 SNP in the Bosnian subjects with non-stricturing non-penetrating CD (B1) compared to cases with stricturing and/or penetrating disease behavior (B2 and/or B3). The association with complications occurrence was also found in the Polish population for rs1004819: A variant, as it was significantly less frequent in subjects with non-stricturing non-penetrating CD (B1) compared to those with more severe disease behavior (B2 or B3). Simultaneously, the MAF of rs1004819 SNP was significantly higher in the Polish subjects with penetrating CD (B3) compared to subjects with non-stricturing non-penetrating or stricturing disease phenotype (B1 or B2). All in all, these results suggest that *IL23R* SNPs could affect CD clinical manifestation. To date, only a small number of studies investigated genotype-phenotype associations of the *IL23R* rs1004819 and 7517847 SNPs in CD and reported results were contradictory. The *IL23R* rs10048419 association with the disease location was firstly described by Glas et al. They have found that ileal disease location (L1 or L3) was significantly more frequent in German patients with rs1004819: A/A (T/T) genotype than in those with rs1004819: G/G (C/C) genotype. At the same time, colonic disease location (L2) was significantly less frequent in subjects with rs1004819: A/A (T/T) genotype compared to carriers of the rs1004819: G/G (C/C) genotype. The increased incidence of structuring disease behavior (B2) was found in group of rs1004819: A/A (T/T) homozygotes compared to carriers of the rs1004819 G/A (C/T) genotype [34]. For the rs7517847 SNP, its minor allele was linked to decreased risk of disease development between 17 and 40 years of life in the New Zealand population, as rs7517847: G allele was significantly less frequent in subjects diagnosed in this particular age group (A2) compared to controls. The rs7517847: G allele was also found to be associated with decreased risk of colonic (L2) and ileo-colonic (L3) disease location, as well as with decreased risk of structuring (B2) and penetrating (B3) CD behavior. Furthermore, in the New Zealand population the minor allele of rs7517847 was associated with a significantly decreased risk of structuring disease with ileal involvement [32]. Additionally, the *IL23R* rs7517847 association with CD location was reported by Doecke et al. in the combined Australian-New Zealand cohort. They have found that the rs7517847: G allele frequency was significantly lower in individuals with colonic (L2) than ileal (L1) CD location. It was also showed that the rs7517847: G variant is significantly less frequent in groups of patients with ileal (L1) as well as with colonic (L2) or ileo-colonic disease location (L3) compared to controls [33]. On the other hand, no associations between both *IL23R* SNPs and CD phenotype were found in the Spanish [22] and British populations [35]. No associations were also reported for rs7517847 SNP in the Italian [42] and another Spanish study [43].

A major limitation of the presented study is the small sample size of the investigated groups from both populations, the Bosnian group in particular. Thus, we will re-evaluate this results in subsequent studies with larger sample size of individuals from Polish and Bosnian populations. Nevertheless, it is worth mentioning that genotype and allele frequencies of both investigated *IL23R* SNPs observed in our study are comparable to those reported in above mentioned studies from European-descent populations [22,32,33,34,35,36,37,38,39,40,41,42,43,44,45].

## 5. Conclusions

Our results confirm the *IL23R* SNPs: rs1004819 and rs7517847 contribution to CD susceptibility in the Polish population and suggest their impact on early age of onset and more severe disease course. This is also the first study on rs1004819 and rs7517847 in the Bosnian CD population. A major limitation of the presented study is the small sample size of the investigated groups. Therefore, these results will be re-evaluated in subsequent studies with larger sample size of individuals from both populations.

## Figures and Tables

**Table 1:** Characteristic of groups with and without CD.

Total, *n* = 294	Poland, *n* = 234			Bosnia and Herzegovina, *n* = 60	
	CD, *n* = 117	CD with Phenotypic Data, *n* = 99	Controls, *n* = 117	CD with Phenotypic Data, *n* = 30	Controls, *n* = 30
Sex					
Males, *n* (%)	59 (50.43)	51 (51.52)	59 (50.43)	15 (50.00)	14 (46.67)
Females, *n* (%)	58 (49.57)	48 (48.48)	58 (49.57)	15 (50.00)	16 (53.33)
Age (years)					
Mean ± SD	35.15 ± 12.65	34.84 ± 12.18	35.62 ± 12.90	44.07 ± 14.53	61.30 ± 15.21
Min–Max	18–68	18–66	18–68	18–80	25–83
Categories according to montreal classification (2005)					
Age at diagnosis, *n* (%)					
A1 (≤16 y)	–	9 (9.09)	–	1 (3.33)	–
A2 (17–40 y)	–	74 (74.75)	–	7 (23.33)	–
A3 (>40 y)	–	16 (16.16)	–	22 (73.34)	–
Location, *n* (%)					
L1 (terminal ileum)	–	3 (3.03)	–	12 (40.00)	–
L2 (colon)	–	35 (35.35)	–	5 (16.67)	–
L3 (ileocolon)	–	61 (61.62)	–	13 (43.33)	–
L4 + (upper gastrointestinal tract +)	–	6 (6.06)	–	–	–
L4 − (upper gastrointestinal tract −)	–	93 (93.94)	–	–	–
Behaviour, *n* (%)					
B1 (non-stricturing non-penetrating)	–	54 (54.55)	–	15 (50.00)	–
B2 (stricturing)	–	23 (23.23)	–	8 (26.67)	–
B3 (penetrating)	–	22 (22.22)	–	6 (20.00)	–
B2 + B3 (structuring and penetrating)	–	–	–	1 (3.33)	–
p + (perianal disease +)	–	14 (14.14)	–	4 (13.33)	–
p − (perianal disease −)	–	85 (85.86)	–	26 (86.67)	–

CD—Crohn’s disease; SD—standard deviation.

**Table 2:** Genotype and allele frequencies and case-control associations for *IL23R* SNPs.

*IL23R* Polymorphism	Poland, *n* = 234					Bosnia and Herzegovina, *n* = 60				
	CD, *n* = 117	Controls, *n* = 117	χ^2^ (df)	*p*	OR (95% CI)	CD, *n* = 30	Controls, *n* = 30	χ^2^ (df)	*p*	OR (95% CI)
rs1004819 G > A										
Genotype, *n* (%)										
G/G	44 (37.6)	66 (56.4)	9.35 (2)	**0.0093** *	–	17 (56.7)	16 (53.4)	0.53 (2)	0.7671*	–
G/A	56 (47.9)	43 (36.8)				11 (36.7)	13 (43.3)			
A/A	17 (14.5)	8 (6.8)				2 (6.6)	1 (3.3)			
Genetic model										
Co-dominant										
G/G	44 (37.6)	66 (56.4)	–	–	1.00	17 (56.7)	16 (53.4)	–	–	1.00
A/A	17 (14.5)	8 (6.8)	–	**0.0142**	3.19 (1.27–8.02)	2 (6.6)	1 (3.3)	–	1.0000	1.88 (0.16–22.85)
G/G	44 (37.6)	66 (56.4)	–	–	1.00	17 (56.7)	16 (53.4)	–	–	1.00
G/A	56 (47.9)	43(36.8)	–	**0.0188**	1.95 (1.13–3.39)	11 (36.7)	13 (43.3)	–	0.7901	0.80 (0.28–2.29)
Dominant										
G/G	44 (37.6)	66 (56.4)	–	–	1.00	17 (56.7)	16 (53.4)	–	–	1.00
A/A + G/A	73 (62.4)	51 (43.6)	–	**0.0058**	2.15 (1.27–3.62)	13 (43.3)	14 (46.6)	–	1.0000	0.87 (0.32–2.42)
Recessive										
G/A + G/G	100 (85.5)	109 (93.2)	–	–	1.00	28 (93.4)	29 (96.7)	–	–	1.00
A/A	17 (14.5)	8 (6.8)	–	0.0889	2.32 (0.96–5.60)	2 (6.6)	1 (3.3)	–	1.0000	2.07 (0.18–24.16)
Allele *n* (%)										
G	144 (61.5)	175 (74.8)	–	–	1.00	45 (75.0)	45 (75.0)	–	–	1.00
A	90 (38.5)	59 (25.2)	–	**0.0028**	1.85 (1.25–2.75)	15 (25.0)	15 (25.0)	–	1.0000	1.00 (0.44–2.29)
rs7517847 T > G										
Genotype, *n* (%)										
T/T	55 (47.0)	38 (32.5)	8.71 (2)	**0.0129** *	–	10 (33.3)	7 (23.3)	–	0.1614 *	–
T/G	53 (45.3)	57 (48.7)				13 (43.4)	20 (66.7)	3.61 (2)		
G/G	9 (7.7)	22 (18.8)				7 (23.3)	3 (10.0)	–		
Genetic model										
Co-dominant										
T/T	55 (47.0)	38 (32.5)	–	–	1.00	10 (33.3)	7 (23.3)	–	–	1.00
G/G	9 (7.7)	22 (18.8)	–	**0.0064**	0.28 (0.12–0.68)	7 (23.3)	3 (10.0)	–	0.6919	1.63 (0.31–8.61)
T/T	55 (47.0)	38 (32.5)	–	–	1.00	10 (33.3)	7 (23.3)	–	–	1.00
T/G	53 (45.3)	57 (48.7)	–	0.1237	0.64 (0.37–1.12)	13 (43.4)	20 (66.7)	–	0.2387	0.46 (0.14–1.50)
Dominant										
T/T	55 (47.0)	38 (32.5)	–	–	1.00	10 (33.3)	7 (23.3)	–	–	1.00
G/G + T/G	62 (53.0)	79 (67.5)	–	**0.0323**	0.54 (0.32–0.92)	20 (66.7)	23 (76.7)	–	0.5675	0.61 (0.20–1.90)
Recessive										
T/G + T/T	108 (92.3)	95 (81.2)	–	–	1.00	23 (76.7)	27 (90.0)	–	–	1.00
G/G	9 (7.7)	22 (18.8)	–	**0.0195**	0.36 (0.16–0.82)	7 (23.3)	3 (10.0)	–	0.2990	2.74 (0.63–11.83)
Allele, *n* (%)										
T	163 (69.7)	133 (56.8)	–	–	1.00	33 (55.0)	34 (56.7)	–	–	1.00
G	71 (30.3)	101 (43.2)	–	**0.0054**	0.57 (0.39–0.84)	27 (45.0)	26 (43.3)	–	1.0000	1.07 (0.52–2.20)

χ^2^—chi-square value; 95% CI—95% confidence interval; CD—Crohn’s disease; df—degrees of freedom; OR—odds ratio; *p*—*p* value in two-sided chi-square test (marked with *) and two-sided Fisher’s exact test; statistically significant *p* values (*p* < 0.05) are bolded.

**Table 3:** Co-occurrence of *IL23R* risk genotypes in CD patients and controls.

**Genotypes Co-Carriage**	**Poland, *n* = 234**					
	**CD, *n* = 117**	**Controls, *n* = 117**	***p*** **(1)**	**OR (95% CI)**	***p*** **(2)**	**χ^2^ (df)**
Homo- and heterozygous risk genotypes, *n* (%)						
0	31 (26.5)	55 (47.0)	–	1.00	**0.0041**	10.98 (2)
1	44 (37.6)	35 (29.9)	0.0128	2.23 (1.19–4.17)		
2	42 (35.9)	27 (23.1)	**0.0034**	2.76 (1.44–5.31)		
Homozygous risk genotypes, *n* (%)						
0	62 (53.0)	79 (67.6)	–	1.00	**0.0444**	6.23 (2)
1	38 (32.5)	30 (25.6)	0.1392	1.61 (0.90–2.89)		
2	17 (14.5)	8 (6.8)	**0.0309**	2.71 (1.10–6.69)		
**Genotypes Co-Carriage**	**Bosnia and Herzegovina, *n* = 60**					
	**CD, *n* = 30**	**Controls, *n* = 30**	***p*** **(1)**	**OR (95% CI)**	***p*** **(2)**	**χ^2^ (df)**
Homo- and heterozygous risk genotypes, *n* (%)						
0	15 (50.0)	13 (43.3)	–	1.00	0.1943	3.28 (2)
1	7 (23.3)	13 (43.3)	0.2489	0.47 (0.14–1.52)		
2	8 (26.7)	4 (13.4)	0.5048	1.73 (0.42–7.11)		
Homozygous risk genotypes, *n* (%)						
0	20 (66.7)	23 (65.0)	–	1.00	0.6609	0.83 (2)
1	8 (26.7)	6 (30.0)	0.5497	1.53 (0.45–5.18)		
2	2 (6.6)	1 (5.0)	0.6000	2.30 (0.19–27.32)		

χ^2^—chi-square value; 95% CI—95% confidence interval; CD—Crohn’s disease; df—degrees of freedom; OR—odds ratio; *p* (1)—*p* value in two-sided Fisher’s exact test; *p* (2)—*p* value in two-sided chi-square test; homo- and heterozygous risk genotypes: rs1004819: A/A or G/A and rs7517847: G/G; homozygous risk genotypes: rs1004819: A/A and rs7517847: G/G; statistically significant *p* values (*p* < 0.05) are bolded.

## Supplementary Materials

The following are available online at https://www.mdpi.com/1660-4601/16/9/1551/s1, Table S1: Genotype and allele frequencies in subgroups of the Polish and Bosnian CD patients and association between genetic variants of *IL23R* and CD phenotype.
